# Comparison of actionable events detected in cancer genomes by whole-genome sequencing, *in silico* whole-exome and mutation panels

**DOI:** 10.1016/j.esmoop.2022.100540

**Published:** 2022-07-15

**Authors:** P. Ramarao-Milne, O. Kondrashova, A.-M. Patch, K. Nones, L.T. Koufariotis, F. Newell, V. Addala, V. Lakis, O. Holmes, C. Leonard, S. Wood, Q. Xu, P. Mukhopadhyay, M.M. Naeini, D. Steinfort, J.P. Williamson, M. Bint, C. Pahoff, P.T. Nguyen, S. Twaddell, D. Arnold, C. Grainge, F. Basirzadeh, D. Fielding, A.J. Dalley, H. Chittoory, P.T. Simpson, L.G. Aoude, V.F. Bonazzi, K. Patel, A.P. Barbour, D.A. Fennell, B.W. Robinson, J. Creaney, G. Hollway, J.V. Pearson, N. Waddell

**Affiliations:** 1 Cancer Program, QIMR Berghofer Medical Research Institute, Brisbane, Australia; 2 Australian e-Health Research Centre, Commonwealth Scientific and Industrial Research Organisation, Brisbane, Australia; 3 Department of Thoracic Medicine, Royal Melbourne Hospital, Melbourne, Australia; 4 Department of Thoracic Medicine, Liverpool Hospital Sydney, Sydney, Australia; 5 Department of Thoracic Medicine, Sunshine Coast University Hospital, Birtinya, Australia; 6 Department of Respiratory Medicine, Gold Coast University Hospital, Southport, Australia; 7 Department of Thoracic Medicine, Royal Adelaide Hospital, Adelaide, Australia; 8 Department of Respiratory and Sleep Medicine, John Hunter Hospital, Newcastle, Australia; 9 Department of Thoracic Medicine, Royal Brisbane and Women’s Hospital, Brisbane, Australia; 10 UQ Centre for Clinical Research, Faculty of Medicine, University of Queensland, Brisbane, Australia; 11 The University of Queensland Diamantina Institute, Faculty of Medicine, University of Queensland, Brisbane, Australia; 12 Upper Gastro-intestinal Surgical Unit, Department of Surgery, Princess Alexandra Hospital, Brisbane, Australia; 13 Cancer Research UK Centre Leicester, University of Leicester & University Hospitals of Leicester NHS Trust, Leicester, UK; 14 National Centre for Asbestos Related Disease, Institute of Respiratory Health, University of Western Australia, Nedlands, Australia; 15 Department of Respiratory Medicine, Sir Charles Gairdner Hospital, Nedlands, Australia

**Keywords:** clinical genomics, whole-genome sequencing, cancer genomics, actionable mutations, tumour mutation burden (TMB), microsatellite instability, precision oncology

## Abstract

**Background:**

Next-generation sequencing is used in cancer research to identify somatic and germline mutations, which can predict sensitivity or resistance to therapies, and may be a useful tool to reveal drug repurposing opportunities between tumour types. Multigene panels are used in clinical practice for detecting targetable mutations. However, the value of clinical whole-exome sequencing (WES) and whole-genome sequencing (WGS) for cancer care is less defined, specifically as the majority of variants found using these technologies are of uncertain significance.

**Patients and methods:**

We used the Cancer Genome Interpreter and WGS in 726 tumours spanning 10 cancer types to identify drug repurposing opportunities. We compare the ability of WGS to detect actionable variants, tumour mutation burden (TMB) and microsatellite instability (MSI) by using *in silico* down-sampled data to mimic WES, a comprehensive sequencing panel and a hotspot mutation panel.

**Results:**

We reveal drug repurposing opportunities as numerous biomarkers are shared across many solid tumour types. Comprehensive panels identify the majority of approved actionable mutations, with WGS detecting more candidate actionable mutations for biomarkers currently in clinical trials. Moreover, estimated values for TMB and MSI vary when calculated from WGS, WES and panel data, and are dependent on whether all mutations or only non-synonymous mutations were used. Our results suggest that TMB and MSI thresholds should not only be tumour-dependent, but also be sequencing platform-dependent.

**Conclusions:**

There is a large opportunity to repurpose cancer drugs, and these data suggest that comprehensive sequencing is an invaluable source of information to guide clinical decisions by facilitating precision medicine and may provide a wealth of information for future studies. Furthermore, the sequencing and analysis approach used to estimate TMB may have clinical implications if a hard threshold is used to indicate which patients may respond to immunotherapy.

## Highlights

• Genome analysis revealed that treatment biomarkers are shared across solid tumours, highlighting repurposing opportunities.
• Comprehensive panels detect most known biomarkers; however, WGS detects more biomarkers for treatments in clinical trials.
• TMB is well correlated between sequencing methods, but absolute values vary and are dependent on mutation types considered.

## Introduction

Comprehensive genomic profiling of tumours provides insight into genomic changes that drive tumourigenesis. International consortia such as The Cancer Genome Atlas (TCGA) and the International Cancer Genome Consortia (ICGC)^1^^,^^2^ conduct whole-genome sequencing (WGS) and whole-exome sequencing (WES) of large cohorts of tumour samples, revealing genes,^3^ non-coding events^4^ and mutational processes^5^ that drive cancer. As our knowledge of cancer genomics accumulates, there are increased opportunities to enable precision medicine, whereby a patient’s tumour genome can be used to predict response or resistance to available drugs. In support of this, the Food and Drug Administration (FDA) has recently approved drugs targeting genomic features of a tumour rather than the tissue of origin.^6^^,^^7^ Ideally, comprehensive sequencing of a patient’s tumour may reveal biomarkers of response or resistance for on-label drugs, or drugs currently approved or under investigation in another indication.

In a clinical setting, genomics is being used to identify tumour-specific somatic and germline mutations that indicate response to targeted therapies. Somatic mutation testing using hotspot mutation or comprehensive gene panels (CGPs) provides rapid, cost-efficient identification of mutations that may inform clinical decisions for approved therapies. The benefits of a panel include small data volume, the ability to profile degraded DNA from formalin-fixed paraffin-embedded (FFPE) tumour samples and cost-effective deep sequencing to allow profiling of samples with low tumour purity or the identification of sub-clonal mutations. The major limitation of panel assays is the requirement of a priori knowledge to select the genes or genomic regions to be assayed.

WES is another cost-effective approach that enables relatively deep sequencing of the coding genome, which is advantageous for profiling samples with low tumour content and for detecting sub-clonal mutations. However, WES has several drawbacks: exome enrichment kits can introduce various artefacts and biases,^8^ affecting copy number (CN) calling,^9^ although the performance of newer tools has reduced these issues significantly.^10^ Additionally, chromosome rearrangements resulting in fusion genes can be missed by WES, when chromosome breakpoints fall outside of exonic regions.^11^ The most comprehensive form of next-generation sequencing is WGS, which provides a relatively even coverage to identify variants across the genome, including non-coding regions, and detects complex genomic rearrangements. However, there are several challenges to the adoption of WGS in the clinic, such as large data volume. Previous studies have compared WGS and WES in both paediatric cancers^12^ and germline sequencing in Mendelian disease^13^ and found that WES failed to detect indels and single-nucleotide variants (SNVs) at specific regions identified in WGS and that WES had a higher false-positive call rate than WGS.

In this study, we explore potential opportunities for drug repurposing in a range of cancer types. Additionally, through *in silico* down-sampling of WGS data, we compare the ability of CGPs, WES and WGS to identify therapeutic targets and call tumour mutation burden (TMB) and microsatellite instability (MSI).

## Patients and methods

### Whole-genome datasets

We compiled 10 WGS datasets with a total of 726 samples, comprising primary tumours from high-grade serous ovarian cancer^14^ (*n* = 76), familial breast cancer^15^ (*n* = 77), breast cancer from TCGA^16^ (*n* = 98), cutaneous melanoma^17^ (*n* = 97), mucosal melanoma^18^ (*n* = 49), pancreatic ductal adenocarcinoma (PDAC)^19^^,^^20^ (*n* = 133), pancreatic neuroendocrine tumour (PNET)^21^ (*n* = 93), oesophageal adenocarcinoma^22^ (*n* = 45), mesothelioma^23^ (*n* = 49) and lung adenocarcinoma (*n* = 9) (Supplementary Table S1, available at https://doi.org/10.1016/j.esmoop.2022.100540).

### Collection and preparation of lung cancer samples

Patients presented with a high pre-test likelihood of a malignant mediastinal or hilar lymph node and underwent endobronchial ultrasound-guided transbronchial needle aspiration for formal diagnosis of lung cancer and for molecular testing. A sample of the aspirate was collected in RNAlater or was snap frozen for research purposes from consenting patients. Institutional Review Board from the Royal Brisbane and Women’s Hospital granted approval for the collection and use of samples (HREC/17/QRBW/301), ratified by other institutes involved in the study. DNA was extracted from the research specimen using the AllPrep DNA/RNA Mini Kit (Qiagen, Australia) and from a blood sample using the QIAamp DNA Blood Mini Kit (Qiagen). WGS was carried out using the TruSeq DNA Nano library preparation and 150 bp paired-end, NovaSeq 6000 sequencing to a target read depth of 30x for normal and 60x for tumour samples.

### WGS analysis and down-sampling to simulate WES and panel sequencing

The WGS analysis for all datasets was carried out as previously described.^18^ Sequence data were aligned to GRCh37 using BWA-MEM.^24^ Somatic SNVs and indels were identified using a dual calling strategy of qSNP^25^ and GATK.^26^ Somatic mutations were annotated with their gene consequence using SNPeff.^27^ Copy number alterations (CNAs) were identified using ascatNgs^28^ and structural variants (SVs) with qSV.^22^ To simulate *in silico* WES and panels, the mutations detected by WGS were down-sampled to filter somatic mutations in relevant regions. The TruSeq DNA Exome kit (Illumina, San Diego, CA) regions were used for WES (covering 37 105 146 bases). The TruSeq Amplicon Cancer Panel (Illumina) was used for the hotspot gene panel (HGP) (covering 16 951 bases), and a CGP was included that covered 2 628 876 bases (see Supplementary Table S2, available at https://doi.org/10.1016/j.esmoop.2022.100540, for a bed file of regions targeted by the CGP). The bedtools application (version 2.25.0) was used to extract the regions covered by the exome and panel kits.

### The Cancer Genome Interpreter and repurposing analyses

Somatic mutations were annotated using the Cancer Genome Interpreter (CGI)^29^ to identify somatic mutations with evidence for treatment response. Additionally, as CGI’s Cancer Biomarker Database (CBD) was last updated in February 2018, biomarker–drug pairs approved by the FDA and National Comprehensive Cancer Network (NCCN) were also included from OncoKB.^30^ Somatic SNVs, indels and CNAs with CN ≥6 were considered as amplifications and those with CN <1 were considered homozygous deletions and were annotated using CGI. For SVs, all entries with a consequence predicted to have a loss of function were annotated as ‘deletions’, while intra-intron fusions were excluded due to the unknown significance of this type of variant. Gene fusion events predicted to create a viable gene fusion product were also submitted to CGI. Only ‘complete’ alterations that were also annotated as driver events (alterations that match the specific amino acid change in the gene which constitutes an actionable variant for a specific drug) were included in our analysis; passenger events and non-protein-affecting variants were excluded. Evidence levels specified by CGI include ‘FDA guidelines’, ‘NCCN guidelines’, ‘Late trials’, ‘Early trials’, ‘Case Report’ and ‘Pre-clinical’. For the repurposing analysis, actionable variants were classified as: FDA-approved cancer-specific (on-label), FDA-approved non-cancer-specific (off-label), clinical trials cancer-specific (on-label) or clinical trials non-cancer-specific (off-label).

#### TMB and MSI calculation

To calculate TMB, the total number of somatic SNV and indels for each sample was used to calculate the number of mutations per megabase (Mb) of the genome. For estimation of TMB using the WGS, mutations were divided by 3000 to obtain mutations per Mb. To simulate the *in silico* exome and panel TMB estimation, the total number of SNV and indels within the down-sampled regions was divided by the number of bases covered by only the exome capture kit, comprehensive and HGP regions (37.105146 Mb, 2.628876 Mb and 0.016951 Mb, respectively). To calculate TMB using the non-synonymous mutations only, a filter from Variant Effect Predictor (VEP)^31^ was used to annotate all somatic mutations as synonymous or non-synonymous and then down-sampled as mentioned previously. MSIsensor^32^ was used to predict MSI using the matched tumour-normal bam files as input. For *in silico* WES and panel down-sampling, only the microsatellite regions which fall within the relevant loci were included. Thresholds to classify samples as MSI-high (MSI-H) were used as suggested in the original paper that used endometrial data.^32^

## Results

### Evidence for repurposing potential across various cancer types in the CBD

**Figure 1:**
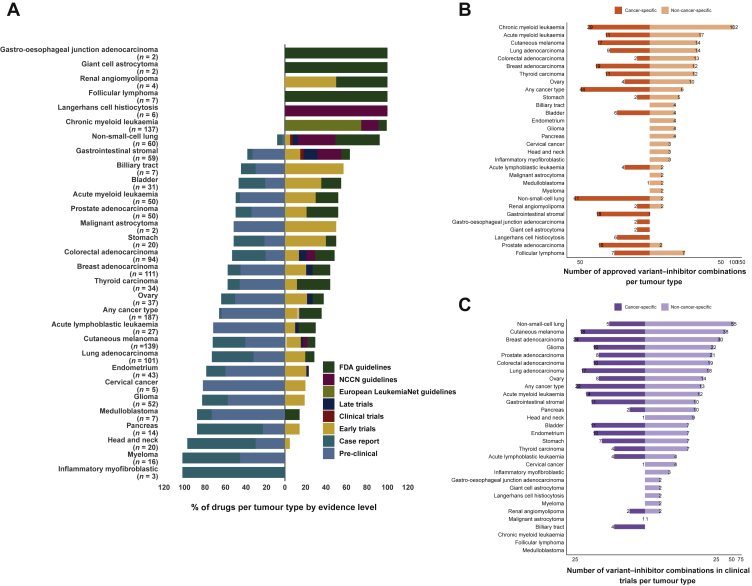
**Overview of drug–biomarker pairs per tumour type within the Cancer Biomarker Database used by the Cancer Genome Interpreter.** (A) Bars which are to the right of the vertical line represent drugs which are either approved or in clinical trials, while bars to the left of the vertical line represent drugs which are either in pre-clinical or case report stages. *n* refers to the number of biomarker–drug combinations for each specified tumour type. (B) Number of biomarkers for which there are cancer type-specific FDA-approved drug allocations for and non-cancer type-specific. (C) Number of biomarkers for which there are cancer type-specific clinical trial drugs for and non-cancer type-specific. FDA, Food and Drug Administration; NCCN, National Comprehensive Cancer Network.

**Supplementary Figure S1:**
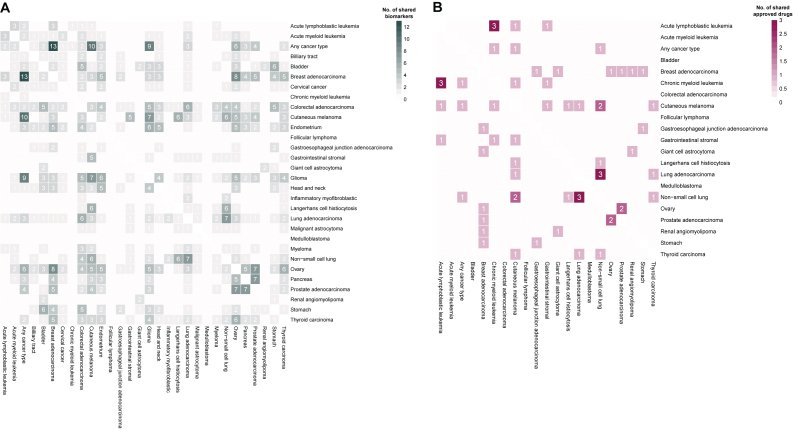
**Supplementary Figure S1: The number of actionable biomarkers in a variety of cancer types.** (A) The number of biomarkers annotated in the cancer biomarker database that are present in and shared between the most tumour types. (B) The number of shared FDA- and NCCN-approved drugs between these solid tumours. GEJ adenocarcinoma, gastro-oesophageal junction adenocarcinoma.

The CBD utilized by CGI contains information about genomic events that have been reported in specific tumours as biomarkers or actionable variants that confer drug sensitivity or resistance. We undertook an analysis of the CBD to survey the biomarker–drug pairs for different tumour types. Some tumour types such as chronic myeloid leukaemia, non-small-cell lung cancer (NSCLC), breast adenocarcinoma and cutaneous melanoma have a large number and percentage of biomarker–drug pairs that are FDA/NCCN approved (Figure 1A and B). In contrast, other tumour types such as endometrial cancer and myeloma primarily only have drugs with pre-clinical or case report-based evidence (Figure 1A). Within the CBD, there are tumour types which lack FDA/NCCN-approved drugs or drugs in clinical trials specific to that tumour type; however, there are mutations with approved drugs in other tumour types. Therefore, we explored the potential of drug repurposing between different tumour types based on shared actionable variants. We found that the number of drugs which could potentially be allocated to a specific tumour based on a specific biomarker can be expanded and includes drugs that have been approved (Figure 1B) or in clinical trials (Figure 1C). Moreover, the number of drugs available for potential repurposing per tumour type increases when drugs in various stages of clinical trials are included (Figure 1C), suggesting that numerous repurposing opportunities exist at the clinical trial level. Within these tumour types, many share a number of genomic biomarkers with each other (Supplementary Figure S1A, available at https://doi.org/10.1016/j.esmoop.2022.100540). However, the number of shared approved drugs is low (Supplementary Figure S1B, available at https://doi.org/10.1016/j.esmoop.2022.100540). Taken together, these data allude to an opportunity to repurpose approved targeted therapies for some tumour types, due to the presence of shared biomarkers.

### Off-label repurposing may offer benefits for patients in some tumour types

**Figure 2:**
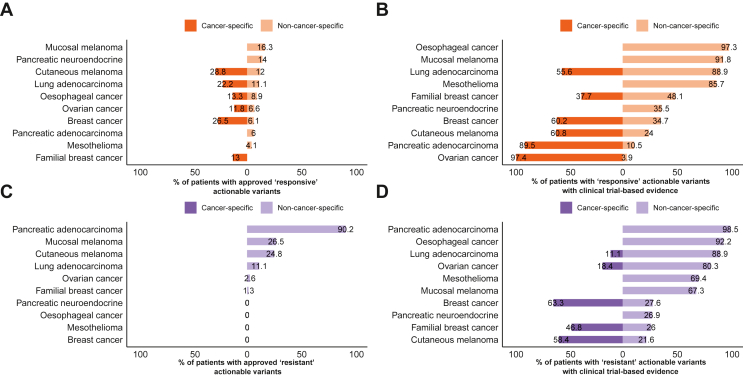
**Repurposing potential of datasets analysed.** Percentage of patients with cancer-specific and non-cancer-specific Food and Drug Administration (FDA)-approved (A) sensitive and (C) resistance biomarkers. Percentage of patients with (B) sensitive and (D) resistance biomarkers for drugs in clinical trials. Bars to the left of *x* = 0 indicate percentage of patients who can be prescribed an on-label drug, bars to the right of *x* = 0 indicate percentage of patients who could be prescribed an off-label drug. Off-label prescriptions are additive to the on-label prescriptions.

To examine the repurposing potential in patient data, we annotated WGS data from 726 tumours from 10 cancer types using CGI. We first sought to determine the percentage of cases that harboured somatic events associated with sensitivity to an approved drug. In the cutaneous melanoma, oesophageal, ovarian, breast, lung adenocarcinoma and familial breast cancer datasets, 28.8%, 13.3%, 11.8%, 26.5%, 22.2% and 13% of cases, respectively, contained biomarkers that indicate cancer-specific on-label prescriptions (Figure 2A). These on-label therapies include BRAF kinase inhibitors such as dabrafenib and vemurafenib in cutaneous melanoma due to the high frequency of *BRAF* mutations, anti-human epidermal growth factor receptor 2 (HER2) therapy such as trastuzumab^33^ for breast and oesophageal cancers based on *ERBB2* amplifications and poly (ADP-ribose) polymerase inhibitors in ovarian and breast cancer based on *BRCA1/2* mutations. Amplification of *ERBB2* or overexpression of the HER2 protein product has been reported in 18%-20% of breast cancers.^34^ We found *ERBB2* amplification in 26.5% of breast cancers and 13% of familial breast cancers (Supplementary Figure S2A, available at https://doi.org/10.1016/j.esmoop.2022.100540); the higher prevalence detected in breast cancer may be due to the threshold we used to define an amplification (of >6 copies with no correction for ploidy), while the lower presence in the familial breast cancers is due to the presence of familial *BRCA*-associated tumours which tend to be HER2 negative. In lung adenocarcinoma, cancer-specific allocations consisted exclusively of epidermal growth factor receptor (*EGFR*) mutations. When off-label drugs were considered, an additional 12%, 11.1%, 8.9%, 6.6% and 6.1% of cutaneous melanoma, lung adenocarcinoma, oesophageal cancer, ovarian cancer and breast cancer cases, respectively, contained biomarkers for targeted therapies.

Conversely, when factoring in putative approved off-label allocations, 16.3% of patients harboured a candidate target including 10.2% of mucosal melanoma cases containing *KIT* mutations, which confer sensitivity to imatinib (Figure 2A). Currently, imatinib is only approved for gastrointestinal stromal tumours with oncogenic *KIT* mutations. In the mucosal melanoma dataset, 4.1% of patients had *BRAF* mutations, predicting sensitivity to BRAF inhibitors. In the PNET dataset, 3.3% of cases harboured *TSC1* or *TSC2* mutations conferring sensitivity to the mammalian target of rapamycin (mTOR) inhibitor, everolimus, currently approved for renal angiomyolipomas and giant cell astrocytomas. The mesothelioma dataset showed a distinct lack of actionable biomarkers. Gene fusions with an approved therapy occurred at a low frequency in all datasets. In the oesophageal cancer dataset, one case was found to have an *NTRK1* fusion, for which entrectinib and larotrectinib are now approved in a solid tumour-agnostic manner. One oesophageal cancer case harboured an *FGFR2* fusion, predicting sensitivity to erdafitinib and pemigatinib, which are approved in cholangiocarcinoma and bladder cancers. Lastly, a PDAC case was found to have an *RET* fusion, for which RET kinase inhibitors such as selpercatinib and pralsetinib are currently approved in NSCLC and thyroid cancer.

When considering variant–drug combinations which are currently in early and late clinical trials, the potential benefit of repurposing is more evident (Figure 2B). Up to 97% of ovarian cancer patients harbour biomarkers for cancer-specific clinical trials, driven primarily by *TP53* mutations. Although targeting *TP53*-mutated cancers is challenging, several tumour-specific clinical trials are currently active for *TP53*-mutated ovarian cancer (NCT02272790 and NCT01164995), both of which assess the efficacy of combination treatment with Wee-1 kinase inhibitors and chemotherapy. Results from one of these clinical trials showed enhanced efficacy of carboplatin in combination with AZD1775 in epithelial ovarian cancer patients who were refractory or resistant to first-line therapy.^35^ Nonetheless, the benefit of therapies in clinical trials is contingent on their proven efficacy, so care must be taken when estimating the repurposing potential of drugs in clinical trials.

Resistance biomarkers primarily consisted of *KRAS* mutations, which confer resistance to anti-EGFR therapies, occurring in 89.5% of PDAC cases, and *NRAS* mutations in the melanoma datasets (Figure 2C, Supplementary Figure S2B, available at https://doi.org/10.1016/j.esmoop.2022.100540). Therefore, cases with sensitivity biomarkers for cetuximab such as EGFR activation will require consideration for the presence of resistance biomarkers that may preclude the use of cetuximab. Similar to responsive markers, the number of patients with biomarkers indicating resistance to treatment was higher when considering clinical trial evidence (Figure 2D).

Taken together, these data show that although some tumour types such as mucosal melanoma may strongly benefit from off-label repurposing of approved therapies, others such as PNET and mesothelioma may remain challenging to treat with currently available targeted therapies. Additionally, while currently available approved drugs may not benefit certain tumour types, this may change if targeted therapies currently in clinical trials are approved in future.

### The percentage of patients with an actionable variant identified by each platform

**Figure 3:**
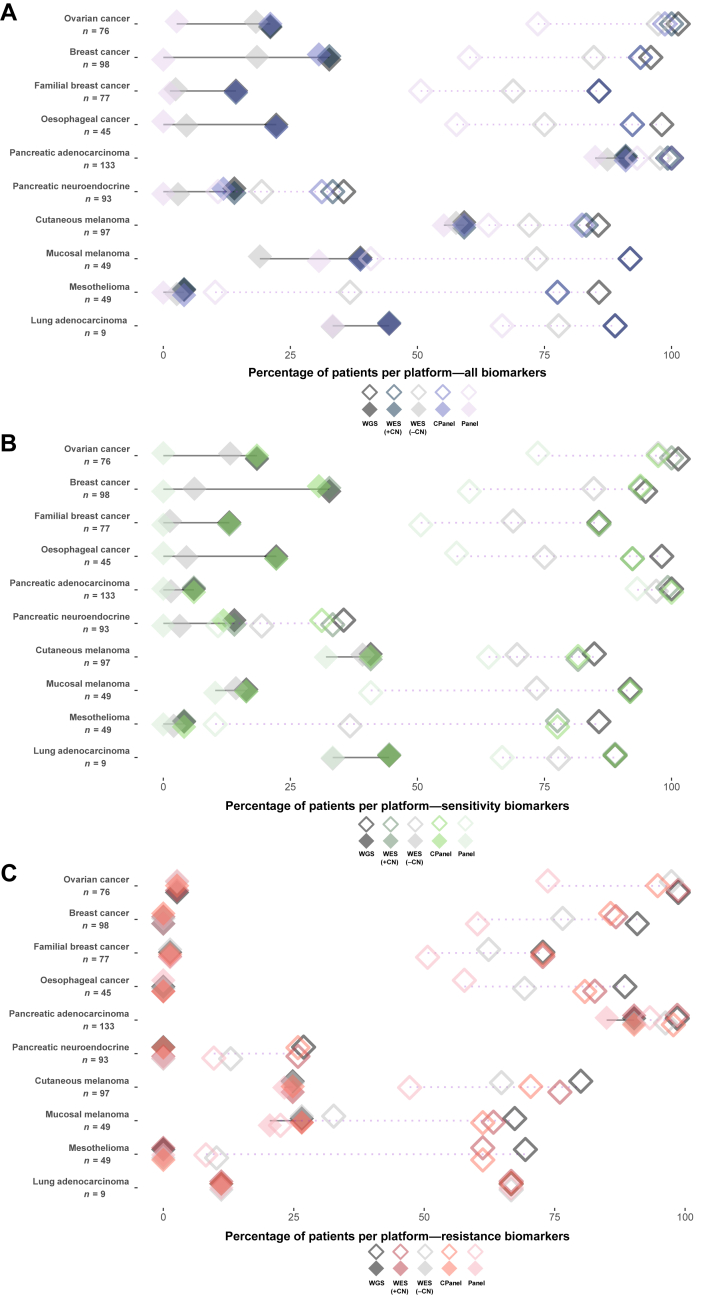
**Comparison of sequencing platforms for the detection of actionable variants in cancer datasets analysed.** Percentage of patients identified by the Cancer Genome Interpreter (CGI) as having (A) actionable variants, (B) actionable variants conferring drug sensitivity and (C) variants conferring drug resistance, stratified by sequencing platform. WES, CPanel and Panel represent *in silico* down-sampled regions of the exome capture kit, comprehensive panel and hotspot mutation panel kit. Solid diamonds joined by solid lines represent percentage of patients with variants for approved drugs only, and open diamonds with dashed lines represent percentage of patients with variants for drugs which are in clinical trials. Drug allocations used are non-cancer-specific (off-label). CN, copy number; CPanel, comprehensive panel; Panel, hotspot panel; WES, whole-exome sequencing; WGS, whole-genome sequencing.

To determine the ability of WGS, WES and panel sequencing to detect actionable mutations, we filtered mutations from the WGS data to select those that fall within the capture of WES, a CGP and an HGP and annotated the data using CGI. In this analysis, we assumed that the HGP could not detect CNAs, and that only WGS and CGP were able to detect gene fusions. Overall, these data revealed that for approved therapies, the HGP, CGP, WES and WGS approaches perform well for the detection of actionable variants in most tumour types (Figure 3A-C, Supplementary Table S3, available at https://doi.org/10.1016/j.esmoop.2022.100540). However, when considering drug–biomarker combinations with clinical trial evidence, WGS identified more patients with biomarkers than other platforms. The higher number of patients with an actionable mutation from WGS was driven by the inclusion of SVs causing gene fusions or a predicted gene loss-of-function events. In terms of actionable SNV and indel variants, WES identified all but three approved actionable markers detected by WGS. The three variants were of low pre-clinical evidence and consisted of a splice acceptor variant and two splice donor variants in *MLL2* (Supplementary Table S4, available at https://doi.org/10.1016/j.esmoop.2022.100540). Mesothelioma had the fewest patients with actionable variants, which may be due to its unusual genomic landscape lacking oncogenic mutations and characterized predominantly by loss of tumour suppressor genes.^36^ Cutaneous melanoma and lung adenocarcinoma contained the largest number of patients with an approved actionable marker detected by all sequencing types which was due to the high prevalence of *BRAF* V600 hotspot and *EGFR* mutations, respectively.

### Prediction of TMB and MSI by WGS and in silico WES and panel sequencing

**Figure 4:**
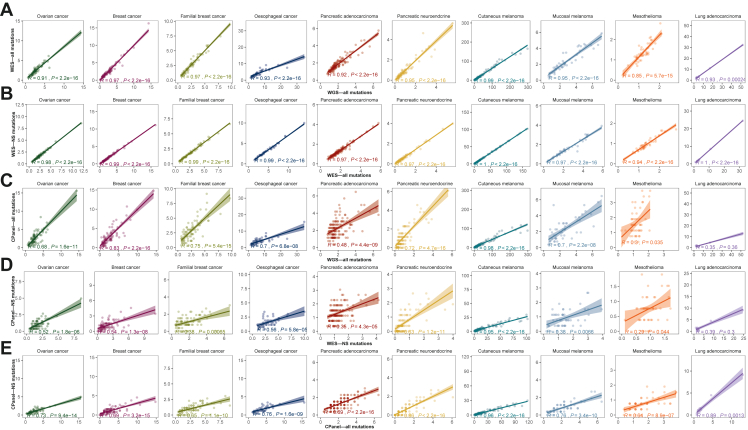
**Estimation of TMB by WGS, *in silico* WES, comprehensive and hotspot panels for 10 tumour types.** (A) TMB calculated using all mutations. Values along the *x*-axis represent the TMB estimated using WGS data, and values along the *y*-axis represent the TMB estimated using *in silico* WES. (B) TMB correlation between *in silico* WES (all mutations) and *in silico* WES (non-synonymous mutations only). (C) TMB correlation between WGS and comprehensive panel (all mutations). (D) TMB correlation between WES and comprehensive panel (non-synonymous mutations only). (E) TMB correlation between comprehensive panel (non-synonymous mutations only) and comprehensive panel (all mutations). CPanel, comprehensive panel; NS, non-synonymous; TMB, tumour mutation burden; WES, whole-exome sequencing; WGS, whole-genome sequencing.

Immune checkpoint blockade therapies such as those targeting programmed cell death protein 1 (PD-1) and cytotoxic T-lymphocyte–associated antigen 4 are effective for the treatment of skin, lung, mesothelioma, bladder and kidney cancers.^37^^,^^38^ Immunohistochemistry of programmed death-ligand 1 is used as a predictive biomarker for immune checkpoint therapy; however, other suggested genomic markers of immunotherapy response are high TMB or MSI.^39^ We calculated the TMB and MSI from the genomic data within the 10 tumour cohorts. As expected, tumours with the highest TMB were cutaneous melanoma, while some cases within tumour types that classically show lower TMB and MSI, such as ovarian cancer, contained a subset of cases that exhibited high TMB (TMB >10 mutations/Mb) (Figure 4A and Supplementary Table S1, available at https://doi.org/10.1016/j.esmoop.2022.100540) and MSI (Supplementary Figure S3 and Table S1, available at https://doi.org/10.1016/j.esmoop.2022.100540). As expected, TMB estimated from WES showed a very strong correlation with WGS when considering all mutations; however, the absolute TMB value tended to be lower in WES for some cancer types (Figure 4A). For WES, we observed a strong correlation between the TMB estimated from all coding mutations and non-synonymous mutations (Figure 4B).

The CGP used in our study targets 2.62 Mb; therefore, we compared TMB estimations of the CGP with WES and WGS. We found that in tumour types with a high number of mutations, such as cutaneous melanoma, the correlation between WGS and the CGP was very strong (*R* = 0.98) (Figure 4C). However, when the number of total mutations within the datasets was low, the correlation weakened. In particular, the mesothelioma (*R* = 0.25) and PDAC (*R* = 0.48) datasets showed poor agreeability between TMB estimated from WGS and CGP. This was also reflected when comparing TMB estimations using non-synonymous mutations between WES and CGP (Figure 4D) and between non-synonymous and all mutations in the CGP (Figure 4E), suggesting that this trend does not improve when selecting non-synonymous mutations only. In almost all tumour types, there was a strong correlation between TMB predicted by WGS, *in silico* WES and *in silico* CGP. Furthermore, when a threshold of 10 mutations/Mb was used, eight oesophageal cancer samples and nine cutaneous melanoma samples exhibited a TMB of >10 for WGS, while showing a TMB <10 for *in silico* WES and CGP (Supplementary Table S1, available at https://doi.org/10.1016/j.esmoop.2022.100540).

MSI estimations were fairly concordant between WGS and WES (Supplementary Figure S3, available at https://doi.org/10.1016/j.esmoop.2022.100540), with the exception of two familial breast cancer cases, a PDAC case and a cutaneous melanoma that were classified as microsatellite-stable (MSS) by WES and MSI-high by WGS. Conversely, there was only one oesophageal cancer case classified as MSI-H by WES and MSS by WGS. These discordant cases, although rare, might have implications when allocating patients to immunotherapy based on MSI, as using a hard threshold will likely exclude some patients who may benefit from immunotherapy, or result in selection of patients unlikely to respond to immunotherapy.

## Discussion

Genomics-guided cancer treatment requires a large selection of therapies with actionable drug–biomarker combinations to be successful. Here, we show that cross-cancer drug repurposing may offer potential opportunity for a range of solid tumours, especially when considering therapies which are currently in clinical trials. Repurposing drugs is an advantageous approach, as the current costs associated with the synthesis, development and testing of a novel drug are estimated to be around $2-$3 billion.^40^ On the other hand, the cost of repurposing currently available drugs is estimated around $300 million.^40^ Part of this cost goes towards funding clinical trials required to develop potential repurposing opportunities into regulatory body-approved clinical practice, as such, future financial backing will be required for the goals of precision medicine to be realized. By filtering somatic mutations detected in WGS data to simulate WES and panels, we show that depending on the approach, the number of patients detected with an actionable variant may differ between tumour types, and the estimation of TMB or MSI values may also vary. Our analysis also supports the need for comprehensive databases and tools which, after variant annotation, can browse through an up-to-date repository of biomarker–drug combinations that are approved or undergoing assessment in clinical trials.

A major challenge for repurposing drugs is whether a drug–biomarker combination will show similar efficacy in different cancer types. In this study, we have assumed that the presence of a genomic biomarker could suggest clinical efficacy across different cancer types; however, this is often not the case in practice. For example, trastuzumab is a monoclonal antibody which was first approved to treat patients with HER2^+^ breast cancer, and was subsequently approved in *ERBB2*-amplified NSCLC^41^ and *HER2*-overexpressing gastric adenocarcinoma.^42^ However, in PDAC, although *ERBB2* is amplified in ∼2% of cases,^43^ in two separate trials, patients with late-stage PDAC treated with trastuzumab in combination with gemcitabine^44^ and capecitabine^45^ showed poor response rates. An additional issue for genomic-guided treatment is the extensive intratumour heterogeneity in some cancers, which may give rise to sub-clones harbouring multiple driver events or the emergence of drug resistance sub-clones. Moreover, co-occurring mutations within a tumour may affect the efficacy of targeted therapy; therefore, it could be suggested that the effectiveness of drug repurposing is contingent on thorough molecular profiling of all targetable driver events within a tumour. Despite these caveats, our analyses do highlight putative actionable biomarkers in rare or understudied tumour types that warrant further investigation. Together, this emphasizes the need for robust biomarkers and inclusion criteria to determine drug responsiveness to facilitate patient selection and treatment efficacy.

We compared WGS, *in silico* WES and panel approaches for detecting actionable mutations. In terms of detecting approved actionable SNV and indel somatic variants, WGS, WES and the panel approaches performed very comparably. However, WGS was able to detect more patients with a candidate actionable variant that had clinical trial or pre-clinical evidence suggesting that WGS may be useful for patients in a clinical trial setting who lack approved targets. An obvious benefit of WGS is that because the whole genome is sequenced the data can be re-interrogated if new targets are approved; also the data can be used to discover new candidate targets, which may inform future drug development. Additionally, as more knowledge is gained from regions outside the coding genome, we anticipate that there will be more clinical utility from studying the whole genome. In support of this, recent pan-cancer analyses of WGS data^2^ identified non-coding somatic driver events^4^^,^^46^ and somatic SVs associated with regulatory regions that impact gene expression.^47^ The challenges of implementing WGS in the clinic to inform therapy for patients include a higher cost and long turnaround times. Additionally, fresh-frozen tissue is an ideal sample type for WGS, but this presents a major obstacle to effective clinical implementation as tumour tissue samples are commonly prepared as FFPE blocks. The process of fixing tissue in FFPE tissue blocks degrades DNA, which is sub-optimal for WGS and may hinder the identification of somatic mutations and CNA.^48^

There are several limitations to our study. A key limitation is the assumption that WGS, WES and the CGP were able to detect SNV, indel, CNA and SV events with the same sensitivity, but this will not always be the case. For example, WGS may not detect all mutations identified by WES and panel sequencing due to the much larger sequence read depth used in these approaches; similarly, all gene fusion events may not be detectable by the CGP. Additionally, some studies show that identification of CNA from WES can be problematic due to the heterogeneous enrichment of exons, resulting in some regions with very low coverage which may lead to missed or incorrect CNA calls^49^ or may be more prone to batch effects than WGS.^50^ Issues pertaining to uneven coverage have been reported in multiple other studies^13^^,^^51^, ^52^, ^53^, ^54^; however, there have been substantial improvements in library preparation methods to overcome these issues, and bioinformatic tools such as Sequenza have shown high sensitivity and accuracy for CNA detection from WES.^55^ Another assumption made was that the HGP was not able to detect CNAs. The HGP we used in this study was based on the TruSeq panel which is not designed to detect CNA; however, newer targeted panels such as the AmpliSeq™ for Illumina Focus Panel are able to interrogate SNVs, indels and CNAs from 52 genes relevant to multiple solid tumour types. Future studies that undertake sequencing of the same DNA samples using WGS, WES and panels should be conducted to directly compare approaches to identify actionable events and assess the clinical benefit they may provide.

The ability to detect somatic mutations will influence the estimation of TMB. The Cancer Research TMB Harmonization Project is bringing together a team of experts to establish a uniform approach to measure and report TMB across different sequencing panels.^56^ A high TMB may indicate patients who will respond to checkpoint blockade immunotherapy, and as such, the FDA has approved the use of PD-1 inhibitors as a therapy for all solid tumours with a TMB ≥10 mutations/Mb as measured by the FoundationOne CDx assay. Even so, in the literature, the definition of what constitutes a high TMB is not clear, as some studies have suggested that a TMB of >16^57^ has a survival benefit, while others used 10 mutations/Mb as a cut-off.^58^ Reporting of TMB calculations has not been consistent, with some studies using different platforms to estimate TMB.^59^ Within the literature, the approach used to calculate TMB is not uniform, with some studies using all mutations and others using only non-synonymous mutations.^59^ We agree with previous studies reporting that TMB estimations from CGP correlate well with WES and WGS^60^; however, this was only true for some cancer types, as similar to other studies we found that correlations are poor for tumour types with low TMB, even with larger panels.^61^, ^62^, ^63^ Despite TMB correlations being poor in tumour types with low mutation frequency, it can be argued that these tumours are unlikely to respond to immunotherapy and thus may not be clinically relevant. Nonetheless, our results support the need for harmonization of TMB estimations across sequencing platforms, and we suggest caution in the use of TMB thresholds when considering patients for immunotherapy.

In summary, genomics is becoming a cost-effective tool that can enable precision medicine by indicating which drugs may be most suitable for cancer patients. Many drugs that are approved or in testing have been developed for specific cancer types; however, there is a large opportunity to repurpose cancer drugs. Comprehensive sequencing is an invaluable source of information to guide clinical decisions by facilitating precision medicine and provides a wealth of information for future studies.

## References

[1] Hudson T.J., Anderson W., Artez A. (2010). International network of cancer genome projects. Nature.

[2] Campbell P.J., Getz G., Korbel J.O. (2020). Pan-cancer analysis of whole genomes. Nature.

[3] Weinstein J.N., Collisson E.A., Mills G.B. (2013). The Cancer Genome Atlas Pan-Cancer analysis project. Nat Genet.

[4] Rheinbay E., Nielsen M.M., Abascal F. (2020). Analyses of non-coding somatic drivers in 2,658 cancer whole genomes. Nature.

[5] Alexandrov L.B., Nik-Zainal S., Wedge D.C. (2013). Signatures of mutational processes in human cancer. Nature.

[6] Hong D., DuBois S., Kummar S. (2020). Larotrectinib in patients with TRK fusion-positive solid tumours: a pooled analysis of three phase 1/2 clinical trials. Lancet Oncol.

[7] Brahmer J.R., Tykodi S.S., Chow L.Q.M. (2012). Safety and activity of anti-PD-L1 antibody in patients with advanced cancer. N Engl J Med.

[8] Shin S.C., Ahn D.H., Kim S.J. (2013). Advantages of single-molecule real-time sequencing in high-GC content genomes. PloS One.

[9] Kadalayil L., Rafiq S., Rose-Zerilli M.J.J. (2015). Exome sequence read depth methods for identifying copy number changes. Brief Bioinform.

[10] Zhao L., Liu H., Yuan X., Gao K., Duan J. (2020). Comparative study of whole exome sequencing-based copy number variation detection tools. BMC Bioinform.

[11] Fromer M., Moran J.L., Chambert K. (2012). Discovery and statistical genotyping of copy-number variation from whole-exome sequencing depth. Am J Hum Genet.

[12] Rusch M., Nakitandwe J., Shurtleff S. (2018). Clinical cancer genomic profiling by three-platform sequencing of whole genome, whole exome and transcriptome. Nat Commun.

[13] Belkadi A., Bolze A., Itan Y. (2015). Whole-genome sequencing is more powerful than whole-exome sequencing for detecting exome variants. Proc Natl Acad Sci U S A.

[14] Patch A.-M., Christie E.L., Etemadmoghadam D. (2015). Whole–genome characterization of chemoresistant ovarian cancer. Nature.

[15] Nones K., Johnson J., Newell F. (2019). Whole-genome sequencing reveals clinically relevant insights into the aetiology of familial breast cancers. Ann Oncol.

[16] Cancer Genome Atlas Network (2012). Comprehensive molecular portraits of human breast tumours. Nature.

[17] Hayward N.K., Wilmott J.S., Waddell N. (2017). Whole-genome landscapes of major melanoma subtypes. Nature.

[18] Newell F., Kong Y., Wilmott J.S. (2019). Whole-genome landscape of mucosal melanoma reveals diverse drivers and therapeutic targets. Nat Commun.

[19] Bailey P., Chang D.K., Nones K. (2016). Genomic analyses identify molecular subtypes of pancreatic cancer. Nature.

[20] Waddell N., Pajic M., Patch A.M. (2015). Whole genomes redefine the mutational landscape of pancreatic cancer. Nature.

[21] Scarpa A., Chang D.K., Nones K. (2017). Whole-genome landscape of pancreatic neuroendocrine tumours. Nature.

[22] Nones K., Waddell N., Wayte N. (2014). Genomic catastrophes frequently arise in esophageal adenocarcinoma and drive tumorigenesis. Nat Commun.

[23] Creaney J., Patch A.M., Addala V. (2022). Comprehensive genomic and tumour immune profiling reveals potential therapeutic targets in malignant pleural mesothelioma. Genome Med.

[24] Li H. (2013).

[25] Kassahn K.S., Holmes O., Nones K. (2013). Somatic point mutation calling in low cellularity tumors. PloS One.

[26] McKenna A., Hanna M., Banks E. (2010). The Genome Analysis Toolkit: a MapReduce framework for analyzing next-generation DNA sequencing data. Genome Res.

[27] Cingolani P., Platts A., Wang L.L. (2012). A program for annotating and predicting the effects of single nucleotide polymorphisms, SnpEff: SNPs in the genome of Drosophila melanogaster strain w1118; iso-2; iso-3. Fly (Austin).

[28] Raine K.M., van Loo P., Wedge D.C. (2016). ascatNgs: identifying somatically acquired copy-number alterations from whole-genome sequencing data. Curr Protoc Bioinformatics.

[29] Tamborero D., Rubio-Perez C., Deu-Pons J. (2018). Cancer Genome Interpreter annotates the biological and clinical relevance of tumor alterations. Genome Med.

[30] Chakravarty D., Gao J., Phillips S. (2017). OncoKB: a precision oncology knowledge base. JCO Precis Oncol.

[31] McLaren W., Gil L., Hunt S.E. (2016). The Ensembl Variant Effect Predictor. Genome Biol.

[32] Niu B., Ye K., Zhang Q. (2014). MSIsensor: microsatellite instability detection using paired tumor-normal sequence data. Bioinformatics.

[33] Gajria D., Chandarlapaty S. (2011). HER2-amplified breast cancer: mechanisms of trastuzumab resistance and novel targeted therapies. Expert Rev Anticancer Ther.

[34] Petrelli F., Tomasello G., Barni S., Lonati V., Passalacqua R., Ghidini M. (2017). Clinical and pathological characterization of HER2 mutations in human breast cancer: a systematic review of the literature. Breast Cancer Res Treat.

[35] Leijen S., van Geel R.M.J.M., Sonke G.S. (2016). Phase II study of WEE1 inhibitor AZD1775 plus carboplatin in patients with TP53-mutated ovarian cancer refractory or resistant to first-line therapy within 3 months. J Clin Oncol.

[36] Hmeljak J., Sanchez-Vega F., Hoadley K.A. (2018). Integrative molecular characterization of malignant pleural mesothelioma. Cancer Discov.

[37] Baas P., Scherpereel A., Nowak A.K. (2021). First-line nivolumab plus ipilimumab in unresectable malignant pleural mesothelioma (CheckMate 743): a multicentre, randomised, open-label, phase 3 trial. Lancet.

[38] Chalmers Z.R., Connelly C.F., Fabrizio D. (2017). Analysis of 100,000 human cancer genomes reveals the landscape of tumor mutational burden. Genome Med.

[39] Luchini C., Bibeau F., Ligtenberg M.J.L. (2019). ESMO recommendations on microsatellite instability testing for immunotherapy in cancer, and its relationship with PD-1/PD-L1 expression and tumour mutational burden: a systematic review-based approach. Ann Oncol.

[40] Nosengo N. (2016). Can you teach old drugs new tricks?. Nature.

[41] Ettinger D.S., Wood D.E., Aisner D.L. (2017). Non-small cell lung cancer, version 5.2017, NCCN Clinical Practice Guidelines in Oncology. J Natl Compr Canc Netw.

[42] Bang Y.-J., Van Cutsem E., Feyereislova A. (2010). Trastuzumab in combination with chemotherapy versus chemotherapy alone for treatment of HER2-positive advanced gastric or gastro-oesophageal junction cancer (ToGA): a phase 3, open-label, randomised controlled trial. Lancet.

[43] Chou A., Waddell N., Cowley M.J. (2013). Clinical and molecular characterization of HER2 amplified-pancreatic cancer. Genome Med.

[44] Safran H., Iannitti D., Ramanathan R. (2004). Herceptin and gemcitabine for metastatic pancreatic cancers that overexpress HER-2/neu. Cancer Invest.

[45] Harder J., Ihorst G., Heinemann V. (2012). Multicentre phase II trial of trastuzumab and capecitabine in patients with HER2 overexpressing metastatic pancreatic cancer. Br J Cancer.

[46] Rodriguez-Martin B., Alvarez E.G., Baez-Ortega A. (2020). Pan-cancer analysis of whole genomes identifies driver rearrangements promoted by LINE-1 retrotransposition. Nat Genet.

[47] Zhang Y., Chen F., Fonseca N.A. (2020). High-coverage whole-genome analysis of 1220 cancers reveals hundreds of genes deregulated by rearrangement-mediated cis-regulatory alterations. Nat Commun.

[48] Robbe P., Popitsch N., Knight S.J.L. (2018). Clinical whole-genome sequencing from routine formalin-fixed, paraffin-embedded specimens: pilot study for the 100,000 Genomes Project. Genet Med.

[49] Hoischen A., Krumm N., Eichler E.E. (2014). Prioritization of neurodevelopmental disease genes by discovery of new mutations. Nat Neurosci.

[50] The Somatic Mutation Working Group of the SEQC-II Consortium (2019). Achieving reproducibility and accuracy in cancer mutation detection with whole-genome and whole-exome sequencing. bioRxiv.

[51] Kong S.W., Lee I.-H., Liu X., Hirschhorn J.N., Mandl K.D. (2018). Measuring coverage and accuracy of whole-exome sequencing in clinical context. Genet Med.

[52] Meienberg J., Bruggmann R., Oexle K., Matyas G. (2016). Clinical sequencing: is WGS the better WES?. Hum Genet.

[53] Carss K.J., Arno G., Erwood M. (2017). Comprehensive rare variant analysis via whole-genome sequencing to determine the molecular pathology of inherited retinal disease. Am J Hum Genet.

[54] Barbitoff Y.A., Polev D.E., Glotov A.S. (2020). Systematic dissection of biases in whole-exome and whole-genome sequencing reveals major determinants of coding sequence coverage. Sci Rep.

[55] Favero F., Joshi T., Marquard A.M. (2015). Sequenza: allele-specific copy number and mutation profiles from tumor sequencing data. Ann Oncol.

[56] Merino D.M., McShane L.M., Fabrizio D. (2020). Establishing guidelines to harmonize tumor mutational burden (TMB): in silico assessment of variation in TMB quantification across diagnostic platforms: phase I of the Friends of Cancer Research TMB Harmonization Project. J Immunother Cancer.

[57] Gandara D.R., Paul S.M., Kowanetz M. (2018). Blood-based tumor mutational burden as a predictor of clinical benefit in non-small-cell lung cancer patients treated with atezolizumab. Nat Med.

[58] Kowanetz M., Zou W., Shames D. (2017). OA20.01 tumor mutation burden (TMB) is associated with improved efficacy of atezolizumab in 1L and 2L+ NSCLC patients. J Thorac Oncol.

[59] Meléndez B., van Campenhout C., Rorive S., Remmelink M., Salmon I., D’Haene N. (2018). Methods of measurement for tumor mutational burden in tumor tissue. Transl Lung Cancer Res.

[60] Pestinger V., Smith M., Sillo T. (2020). Use of an integrated pan-cancer oncology enrichment next-generation sequencing assay to measure tumour mutational burden and detect clinically actionable variants. Mol Diagn Ther.

[61] Buchhalter I., Rempel E., Endris V. (2019). Size matters: dissecting key parameters for panel-based tumor mutational burden analysis. Int J Cancer.

[62] Endris V., Buchhalter I., Allgäuer M. (2019). Measurement of tumor mutational burden (TMB) in routine molecular diagnostics: in silico and real-life analysis of three larger gene panels. Int J Cancer.

[63] Hatakeyama K., Nagashima T., Urakami K. (2018). Tumor mutational burden analysis of 2,000 Japanese cancer genomes using whole exome and targeted gene panel sequencing. Biomed Res.

