# Supplementary material for: Comparison of actionable events detected in cancer genomes by whole-genome sequencing, in silico whole-exome and mutation panels
Source: ESMO Open. 2022 Jul 15;7(4):100540. doi: 10.1016/j.esmoop.2022.100540 (PMC9463385; doi:10.1016/j.esmoop.2022.100540)
Supplement: Supplementary Figure S2 [file mmc1.pdf]

SUPPLEMENTARY FIGURE 2A

A FDA/NCCN-APPROVED "RESPONSIVE" ACTIONABLE VARIANTS

ONCOGENIC MUTATIONS

	BRAF oncogenic mutation	*BRCA1* oncogenic mutation	*BRCA2* oncogenic mutation	*DNMT3A* oncogenic mutation	*EGFR* oncogenic mutation	*KIT* oncogenic mutation	*NPM1* oncogenic mutation	*PDGFRA* oncogenic mutation	*PTCH1* oncogenic mutation	*TSC1* oncogenic mutation	*TSC2* oncogenic mutation
Cutaneous Melanoma	28.8	2.4	3.2	0.8	0.0	3.2	0	0.8	1.6	2.4	3.2
Lung Adenocarcinoma	0.0	0.0	0.0	0.0	22.2	0.0	0	0.0	0.0	0.0	0.0
Mucosal Melanoma	4.1	0	0	0	0.0	10.2	0	0	0	0	0
Ovarian Cancer	0	7.9	3.9	0	0	0	1.3	0	0	0	0
Breast Cancer	0	2.0	3.1	1.0	0	0	0	0	0	0	0
Oesophageal Cancer	0	0	0	2.2	0	0	0	0	2.2	0	0
Pancreatic Neuroendocrine	0	0	0	0	0	0	0	0	0	1.1	2.2
Mesothelioma	0	2.0	0	0	0	0	0	0	0	0	0
Pancreatic Adenocarcinoma	0	0	1.5	0	0	0	0	0	0	0	0
Familial Breast Cancer	0	1.3	0	0	0	0	0	0	0	0	0

COPY NUMBER ABERRATIONS

	ERBB2 amplification
Breast Cancer	26.5
Oesophageal Cancer	17.8
Familial Breast Cancer	13.0
Lung Adenocarcinoma	11.1
Pancreatic Neuroendocrine	10.8
Ovarian Cancer	5.3
Pancreatic Adenocarcinoma	4.5
Mucosal Melanoma	2.0
Mesothelioma	2.0
Cutaneous Melanoma	1.6

GENE FUSIONS

	FGFR2 fusion	*NTRK1* fusion	*RET* fusion
Oesophageal Cancer	2.2	2.2	0
Pancreatic Adenocarcinoma	0	0	0.8
Breast Cancer	0	0	0
Lung Adenocarcinoma	0	0	0
Familial Breast Cancer	0	0	0
Pancreatic Neuroendocrine	0	0	0
Ovarian Cancer	0	0	0
Mucosal Melanoma	0	0	0
Mesothelioma	0	0	0
Cutaneous Melanoma	0	0	0

B FDA/NCCN-APPROVED "RESISTANT" VARIANTS

ONCOGENIC MUTATIONS

	KIT oncogenic mutation	*KRAS* oncogenic mutation	*NRAS* oncogenic mutation
Pancreatic Adenocarcinoma	0	89.5	0.8
Mucosal Melanoma	2.0	2.0	22.4
Cutaneous Melanoma	0	0.8	24.8
Ovarian Cancer	0	1.3	1.3
Familial Breast Cancer	0	0	1.3
Lung Adenocarcinoma	0	11.1	0
Mesothelioma	0	0	0
Pancreatic Neuroendocrine	0	0	0
Breast Cancer	0	0	0
Oesophageal Cancer	0	0	0
