# Supplementary figures and images for: Comparison of actionable events detected in cancer genomes by whole-genome sequencing, in silico whole-exome and mutation panels
Source: ESMO Open. 2022 Jul 15;7(4):100540. doi: 10.1016/j.esmoop.2022.100540 (PMC9463385; doi:10.1016/j.esmoop.2022.100540)

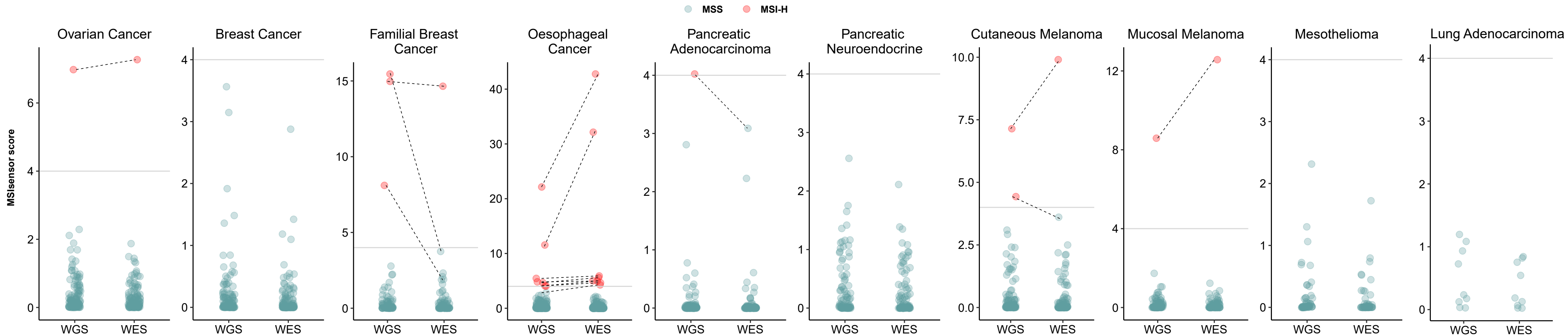

Supplement: Supplementary Figure S3 [file mmc2.pdf]
