# Supplementary material for: Comparison of actionable events detected in cancer genomes by whole-genome sequencing, in silico whole-exome and mutation panels
Source: ESMO Open. 2022 Jul 15;7(4):100540. doi: 10.1016/j.esmoop.2022.100540 (PMC9463385; doi:10.1016/j.esmoop.2022.100540)
Supplement: Supplementary Materials [file mmc7.docx]

**Supplementary Figures**

**Supplementary Figure S1: The number of actionable biomarkers in a variety of cancer types. A**, The number of biomarkers annotated in the cancer biomarker database that are present in and shared between the most tumour types. **B**, The number of shared FDA- and NCCN-approved drugs between these solid tumours. GEJ adenocarcinoma, gastro-oesophageal junction adenocarcinoma.

**Supplementary Figure S2: Percentage of patients in each dataset with actionable variants. A**, “responsive” actionable variants conferring drug sensitivity, and **B**, “resistant” variants. The variants are stratified by nature of genomic aberration: oncogenic mutations, copy number aberrations and gene fusions.

**Supplementary Figure S3: Comparison of microsatellite instability (MSI) estimations in 10 cancer types from WGS and WES.** MSIsensor was used to estimate MSI using the WGS data and in silico WES. A threshold of 4% (grey line) was used to identify MSI-H samples (red dots) in each tumour type.

**Supplementary Tables**

**Supplementary Table S1:** Sample information for the 726 samples in the study and tumour mutation burden (TMB) estimations by WGS, in silico WES and panel assays.

**Supplementary Table S2:** Genome regions targeted by the comprehensive cancer panel used in this study

**Supplementary Table S3.** Analysis of actionable events in 726 patients using the Cancer Genome Interpreter.

**Supplementary Table S4:** Actionable SNVs that were identified in WGS but may be absent from other sequencing platforms.
